# New dedicated blunt straight needles and sutures for uterine compression sutures: a retrospective study and literature review

**DOI:** 10.1186/s12893-019-0495-7

**Published:** 2019-03-11

**Authors:** Shinya Matsuzaki, Masayuki Endo, Takuji Tomimatsu, Satoshi Nakagawa, Satoko Matsuzaki, Tatsuya Miyake, Tsuyoshi Takiuchi, Aiko Kakigano, Kazuya Mimura, Yutaka Ueda, Tadashi Kimura

**Affiliations:** 1 Department of Obstetrics and Gynecology, Osaka University Graduate School of Medicine, 2-2 Yamadaoka, Suita, Osaka, 565-0871 Japan; 2 Department of Obstetrics and Gynecology, Otemae Hospital, 1-5-34, Otemae, Tyuuouku, Osaka, 540-0008 Japan

**Keywords:** Uterine compression suture, Needle, Blunt, B-Lynch, Hayman suture

## Abstract

**Background:**

We developed a dedicated blunt straight needle with No. 2 polydioxanone sutures (2-Monodiox®) for uterine compression sutures (UCSs) and aimed to assess the outcomes and complication rates of UCSs for postpartum hemorrhage by comparing with commercially available needle and suture types.

**Methods:**

A retrospective analysis was performed between January 2010 and February 2018. During the study period, two types of commercially available sutures and 2-Monodiox® were used. PubMed, MEDLINE, and Scopus databases were searched for English articles published between January 1997 and May 2017 using search terms related to the suture and needle types for UCSs to discuss the dedicated needles and sutures for UCS.

**Results:**

The analysis included 47 cases of UCSs for the uterine body with three suture types (No. 0 polydioxanone, 7 cases; No. 1 poliglecaprone 25, 21 cases; and No. 2 polydioxanone, 19 cases). B-Lynch suture using No. 0 sutures was associated with a significantly lower uterine preservation rate than those with Nos. 1 and 2 sutures (42.9% vs. 95.2 and 89.5%, respectively; *p <* 0.01). A modified Hayman suture technique was performed using 2-Monodiox® sutures, which achieved a similar uterine preservation rate compared with B-Lynch suture using No. 1 poliglecaprone 25 sutures. No patients developed severe complications. The literature review showed that no dedicated sutures have developed for UCSs. Three dedicated needles for UCSs have been developed, and 2-Monodiox® is the first dedicated blunt straight needle for UCSs.

**Conclusion:**

Our data showed that No. 0 sutures should not be used for B-Lynch suture. The uterine preservation rate is similar for 2-Monodiox® with modified Hayman suture and No. 1 poliglecaprone 25 sutures with B-Lynch suture, without the occurrence of severe complications.

**Electronic supplementary material:**

The online version of this article (10.1186/s12893-019-0495-7) contains supplementary material, which is available to authorized users.

## Key message

Our dedicated blunt straight needle with No. 2 polydioxanone sutures (2-Monodiox®) could perform modified Hayman suture with similar uterine preservation rate performed with B-Lynch technique with No. 1 sutures.

## Background

Postpartum hemorrhage (PPH) is an obstetrical emergency occurring in 4–6% of live births [1, 2]. Uterine compression sutures (UCSs) have been used for the management of PPH and for avoiding the need for hysterectomy since the introduction of the B-Lynch suture technique in 1997 [3–6]. Various UCSs technique, such as B-Lynch suture, Hayman suture, Cho suture, Pereira suture, and Matsubara Yano suture, have been developed and reported high uterine preservation rate [4, 7–9]. These studies have shown that UCSs are very important techniques for PPH. Also, various studies have focused on the type of UCSs technique.

On the other hand, studies about dedicated needles and sutures for UCSs are limited, and the differences in the efficacies of a needle and sutures have not been compared. Several studies did not even mention about the needles and sutures [10–12] and Polyglactin 910 and Catgut sutures appears to be used in many studies [13–15]. However, detailed information is difficult to obtain because the literature review about sutures and needles for UCSs have not been reported. Therefore, our study focused on the efficacy of needles and sutures for UCSs.

First, we show detailed information about our dedicated blunt straight needle with No. 2 polydioxanone sutures (2-Monodiox®, Alfresa Pharma Co., Osaka, Japan) specially made for UCSs. Our study is the first report of 2-Monodiox® for UCS. Next, the efficacy of different needles and sutures for UCSs used at our institution were compared.

Here, the analysis was performed to examine the data of UCSs in our institution and to determine the efficacy of different needle and suture types for UCSs, including 2-Monodiox®.

## Methods

### Data retrieval

A retrospective analysis of the clinical outcome of patients with PPH treated using UCSs for the uterine body due to atonic bleeding was performed to assess the efficacy of a dedicated needle and to compare the uterine preservation rate for each suture type. Data were reviewed from the medical records of women who underwent UCSs during cesarean delivery due to atonic bleeding at the Osaka University Hospital, Japan, between January 1, 2010, and February 28, 2018. Women with PPH provided informed consent before undergoing surgery with UCSs. The indications for the use of UCSs have been previously described [5]. In principle, UCSs are only used in cesarean deliveries and when the clinician considers that PPH is persistent, with an estimated blood loss of ≥1500 mL. Therefore, women undergoing UCSs were included in our analysis only if the intraoperative blood loss was > 1500 mL. To control PPH due to atonic bleeding, either B-Lynch sutures, as described by B-Lynch et al. with or without some modification [4], or modified Hayman sutures were used as UCSs [7]. A 4–8 week follow up examination was conducted, and the patients were educated to come to our institution to undergo further follow ups if the patients experienced abnormal symptoms, such as abdominal pain, menstrual disorder, and dysmenorrhea.

### Dedicated needle and suture for UCS

**Fig. 1:**
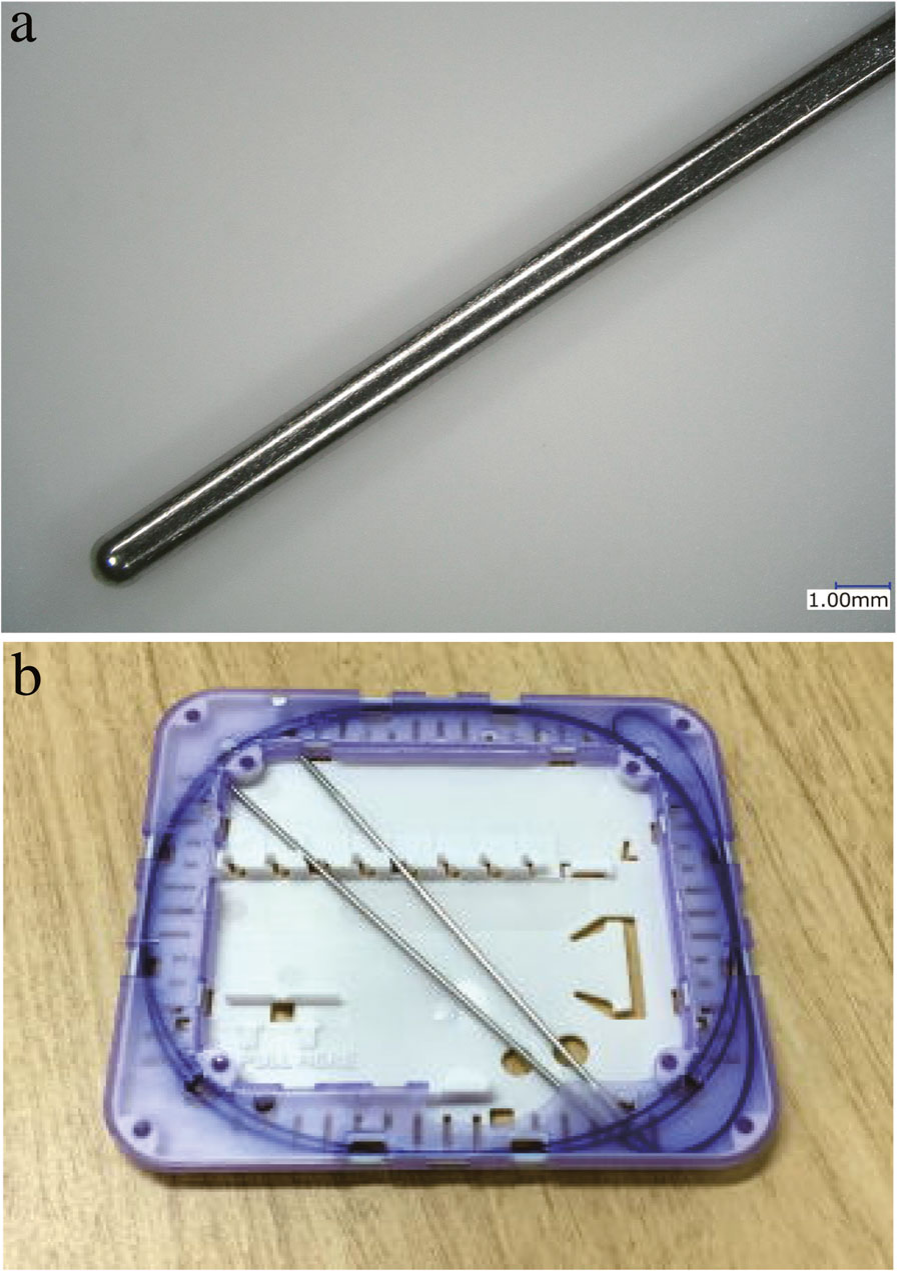
A dedicated blunt straight needle and sutures for UCSs were developed. The lengths of the needle and suture are 80 and 90 cm, respectively. (**a**) The needle was made as blunt as possible to avoid the tearing of the surgeon’s glove, bladder, bowel, and rectum, but to easily penetrate the uterine wall in the anterior to posterior direction. (**b**) The difference in the diameter between the needle and suture was kept minimum, with a final diameter of 0.3 mm to prevent bleeding from the sutured site


To the best of our knowledge, no dedicated straight blunt needle has been developed for UCSs. In Japan, a dedicated 80-mm straight blunt needle was developed by Alfresa Pharma Corporation and Osaka university for use with a 90-cm No. 2 polydioxanone suture (2-Monodiox®). As shown in Fig. 1, the needle tip is extremely blunt and cannot tear the surgeon’s glove but can easily penetrate the uterine wall in the anterior to posterior direction (Additional file 1).

**Additional file 1:** Our method of Modified Hayman suture with 2-Monodiox® sutures. This patient experienced 3050 ml of intraoperative bleeding due to the uterine atony. The surgeon could penetrate uterine wall anterior to posterior easily. Owing to the blunt straight needle and heavy suture, it was easy to perform modified Hayman suture effectively. In this video file, UCS could control the postpartum hemorrhage. (WMV 67341 kb)


### Suture types

We changed the needles and sutures for UCSs thrice to enhance the effectiveness; therefore, three groups based on UCS were formed for analysis. The indications for the use of UCSs in our institution have been mentioned previously [3].

Between January 1, 2010, and August 30, 2013, the B-Lynch suture technique was mainly performed with 48-mm 1/2 circle taper point with 0-PDS® (Z990G; Ethicon Endo-Surgery, Inc., Cincinnati, OH, USA), because the choice of the needle and suture for UCS was limited in our institution (during this period, some surgical records did not describe the suture or needle type used; group A). After September 2013, the B-Lynch technique was performed with a 70-mm 1/2 circle blunt needle and 1-Monocryl® sutures (W3709; Ethicon Endo-Surgery, Inc.), because this needle was not marketed in Japan until June 2013 (group B). In Japan, a dedicated needle (2-Monodiox®) and suture were developed by Alfresa Pharma Corporation (Osaka, Japan) and Osaka University to place UCSs more easily and efficiently. Subsequently, the 2-Monodiox® suture was used to perform the modified Hayman suture technique (group C) from August, 2016.

### Factors

To identify the dedicated needles and sutures for UCSs we performed a literature review. We conducted an electronic search of PubMed (https://www.ncbi.nlm.nih.gov/pubmed/), MEDLINE (https://www.ncbi.nlm.nih.gov), and Scopus (https://www.scopus.com) databases from January 1997 to May 2017. Articles in languages other than English and those published before 1997 were excluded. The search strategy consisted of the following keywords specific to each database: B-Lynch, uterine compression suture, and uterine preserved surgery. In addition, articles related to B-Lynch sutures were included.

### Statistical analysis

JMP Pro version 13.1 software (SAS Institute, Tokyo, Japan) was used to perform statistical analyses. Categorical variables were compared using the Fisher’s exact tests, while continuous variables were compared using the one-way analysis of variance. A probability (*p*) value of < 0.05 was considered statistically significant.

### Ethical approval

The study protocol was approved on July 19, 2017, by the Ethics Committee of Osaka University (approval no. 17129).

## Results

### Surgical outcomes of UCSs with the use of different sutures

Of the 2123 cesarean deliveries during the study period, 162 had estimated blood loss of > 1500 mL due to the atonic bleeding. The B-lynch or modified Hayman suture technique was performed in 47 patients included in this analysis (7 in group A, 21 in group B, and 19 in group C). The patients’ baseline characteristics and surgical outcomes are shown in Tables 1 and 2, respectively. There were no significant differences in patient characteristics among the three groups (Table 1). No patient developed severe complications, such as uterine necrosis and pyometra. There were no significant differences in the endometritis rates among the three groups. The uterine preservation rate was significantly lower in group A than in groups B and C (43.9% vs. 95.2 and 89.5%, respectively; *p* = 0.006; Table 2). There were no significant differences in the uterine preservation rates between groups B and C (*p* = 0.56). No severe complications were detected in the follow up examination, and no patients visited our hospital with abnormal symptoms after the follow up examination.

**Table 1:** Demographic characteristics of women with uterine compression sutures

	Total (*n* = 47)		Group A (*n* = 7)		Group B (*n* = 21)		Group C (*n* = 19)		
	*n*	%	*n*	%	*n*	%	*n*	%	*p* value
Age (years)	37.5 ± 4.5 (25–49)		39.6 ± 5.4 (34–49)		37.4 ± 4.3 (25–45)		36.5 ± 4.3 (27–46)		0.652
< 35	7	17.1	6	85.7	18	85.7	15	78.9	0.870
≥ 35	34	82.9	1	14.3	3	14.3	4	21.1	
Parity									
Primipara	37	78.7	5	71.4	14	66.7	18	94.7	0.07
Multipara	10	22.3	2	28.6	7	33.3	1	5.3	
BMI	23.7 ± 3.0 (18.1–30.9)		23.9 ± 3.8 (19.3–28.3)		23.5 ± 2.7 (18.9–29.2)		23.8 ± 3.1 (18.1–30.9)		0.541
Gestational weeks at delivery	37.8 ± 2.0 (33–41)		37.8 ± 1.6 (36–41)		37.6 ± 2.2 (33–41)		38.0 ± 1.6 (35–41)		0.652
Preterm delivery	9	19.1	0	0	5	23.8	4	21.1	0.531
Birthweight (g)	2945.8 ± 581.5 (1616–4556)		2912.3 ± 240.4 (2548–3278)		2929.9 ± 693.9 (1616–4244)		2971.8 ± 548.2 (2304–4556)		0.976
Indication of cesarean delivery									
Placenta previa	4	8.5	2	28.6	1	4.8	1	5.3	0.193
Previous cesarean delivery	9	19.1	1	14.3	7	33.3	1	5.3	0.078
Breech presentation	2	4.3	1	14.3	0		1	5.3	0.142
Arrest of labor	14	31.9	1	14.3	6	28.6	7	36.8	0.578
Non-reassuring fetal status	4	8.5	1	14.3	2	9.5	1	5.3	0.799
Twins	6	12.8	0	0.0	2	9.5	4	21.0	0.503
Others	7	14.5	1	14.3	2	9.5	4	21.0	
Type of cesarean delivery									
Elective	16	34.0	2	28.5	7	33.3	7	36.8	0.92
Emergent	31	66.0	5	71.5	14	66.7	12	63.2	
Suture type									
B-Lynch	28	57.1	7	100	21	100	0	0	
Modified Hayman	21	42.9	0	0	0	0	21	100	

Data are presented as *n* (%) or mean ± standard deviation, unless otherwise specified

**Table 2:** Postoperative outcomes of women with uterine compression sutures

	All cases (*n* = 41)		Group A (*n* = 7)		Group B (*n* = 21)		Group C (*n* = 19)		
	*n*	%	*n*	%	*n*	%	*n*	%	*p* value
Estimated blood loss (ml)	2358 ± 960 (1500–6600)		3682 ± 1481 (2200–6600)		2110 ± 659 (1500–4000)		2143 ± 960 (1500–3600)		
Transfusion	**22**	**53.6**	**7**	**100**	**10**	**47.6**	**8**	**42.1**	**0.02**
Duration of surgery (min)	111.6 ± 44.1		156.9 ± 41.8		108.2 ± 42.3		98.6 ± 37.4		0.128
Additional treatment									
Balloon tamponade	3	7.3	0	0	1	4.8	3	15.8	0.359
UAE	0	0	0	0	0	0	0	0	1.00
Balloon tamponade →UAE	1	2.4	0	0	0	0	1	5.3	0.553
Hysterectomy	**6**	**14.6**	**4**	**57.1**	**1**	**4.8**	**2**	**10.5**	**0.006**
Postoperative complications									
Endometritis	5	12.2	0	0	3	14.3	2	10.5	0.836
Ileus	6	14.6	0	0	2	9.5	4	21.1	0.503

Data are presented as *n* (%) or mean ± standard deviation, unless otherwise specified

Bold indicates statistical significance

*UAE* Uterine artery embolization

### Needles and sutures used for UCS in previous studies

Studies identified with Pubmed, MEDLINE, and Scopus were 243, 258, and 305, respectively. Seventy six studies mentioned the type of sutures in their studies, and approximately 80% of studies used Polyglactin 910 and Catgut sutures (data not shown). No dedicated suture for UCSs was identified.

Literature review showed that the three dedicated needles including our study were used for UCSs: No. 1 poliglecaprone 25 suture (W3709; Ethicon Endo-Surgery, Inc.), which was developed to perform the B-Lynch technique [16, 17], and a 100 mm custom made needle (AZNJ100N, Akiyama, Tokyo, Japan), as reported by Yano et al., which was developed to perform the MY suture technique [18]. We have summarized the surgical outcome of the dedicated needles and sutures for UCSs (Table 3) [5, 19, 20]. Literature review also showed that 2-Monodiox® is the first dedicated specific blunt straight needle for UCSs. This study is the first to report on the use of this needle and suture type. 2-Monodiox® could perform modified Hayman suture, and a supplementary video file shows this in more detail (Additional file 1).

**Table 3:** Summary of the results of surgical outcome of the dedicated needles for uterine compression sutures

Reference number	Year	Author	Cases	Main indication of UCS	Needle and suture types	Method	Adjunctive hemostatic procedures (without hysterectomy)	Intraoperative blood loss (ml)	Rate of transfusion	Rate of uterine preservation	Severe complication^a^	Subsequent pregnancy
11	2011	Yoong W	11	UA+ PP	No. 1 poliglecaprone 25 with ethiguard needle	Uterine sandwich technique	All cases: IUBT	1500 (Median)	NA	11/11 (100%)	None	None
12	2013	Kaoiean S	24	UA		23 cases: B-Lynch suture 1 case: Hayman suture	6 cases: BUAL	1750 (Median)	15/24 (32.5%)	23/24 (95.8%)	None	None
	2018	Current study	21	UA		Modified B-Lynch suture	1 case: IUBT	2110 (Mean)	10/21 (47.6%)	20/21 (95.2%)	None	None
10	2013	Yano H	1	Placenta accreta	100-mm custom-made needle (Suture was unknown)	MY suture	None	NA	NA	1/1 (100%)	NA	NA
	2018	Current study	19	UA	80-mm straight blunt needle with No. 2 polydioxanone suture	Modified Hayman suture	3 cases: IUBT 1 case: IUBT and UAE	2143 (Mean)	8/19 (42.8%)	17/19 (89.5%)	None	1 case: no complication

^a^Severe complications are defined as need for surgical intervention to treat complications due to uterine compression sutures

*BUAL* Bilateral uterine artery ligation

*IUBT* Intra uterine balloon tamponade

*NA* Not available

*PP* Placenta previa

*UA* Uterinetony

## Discussion

We developed a dedicated suture and needle for UCS and successfully showed the similar uterine preservation rate with the B-Lynch technique with No. 1 sutures in this study. Literature review showed that this analysis is the first to compare the three types of needles and sutures used for UCSs, and the review results could make interesting discussion on the current state of sutures and needles used for UCSs.

A comparison of the efficacy of different needles and sutures used as UCSs in our institution revealed that the B-Lynch technique with No. 1 sutures achieved better outcomes than those with No. 0 sutures. Thus, the B-Lynch technique should not be performed with No. 0 sutures, because the uterine preservation rate was significantly lower. Furthermore, our literature review showed that no previous study with a large number of patients has investigated the B-Lynch technique with No. 0 sutures. Our results also suggested that the kind of needles and sutures might have affected the surgical outcome of UCSs. Although groups B and C showed similar uterine preserve rates, discussion about the needles and sutures was difficult because the comparison was not only between different needles and sutures but between different surgical techniques as well. Further studies, such as comparing the same UCS technique with different sutures and needles, are needed to compare the different type of sutures and needles.

Uterine atony is one of the most common causes of PPH, and a simple and effective procedure is ideal in such an emergent situation [21]. A dedicated needle and heavy suture for UCSs are essential for efficient and effective suture placement. We speculated that a dedicated needle could help clinicians to rapidly perform the Hayman, Cho, and MY suture techniques. Therefore, Osaka university and Alfresa Pharma Corporation developed a specific blunt straight needle with No. 2 polydioxanone sutures. We tried to create extremely blunt needle that can avoid the tearing of the surgeon’s glove, bladder, bowel, and rectum, but can easily penetrate the uterine wall in the anterior to posterior direction. No dedicated straight blunt needle has been available for UCSs, and straightening of a curved needle to place UCSs or using liver needle for UCSs have been reported [22–24]. We considered that straightening a curved needle or using liver needle is not ideal and not safe to perform UCSs. Therefore, we developed a dedicated straight blunt needle to improve the current state of straight needles available for UCSs. We considered that using a straight needle was not necessary for UCSs when performing the B-Lynch technique; however, the Hayman, Cho, and MY techniques have been performed using a straight or liver needle in previous studies, and these procedures may be more accessible once a straight blunt needle is developed [25–27]. Future studies are needed to show the simplicity of this device for use in UCSs. In addition, we expected that the dedicated straight blunt needle for UCS would be useful for placenta previa because performing UCSs toward the lower uterine segment is very difficult compared with the uterine body [28]. Further studies are now ongoing at our institution to show the efficacy and simplicity of 2-Monodiox® for placenta previa.

Although, it is only our opinion, we felt that the modified Hayman suture technique with 2-Monodiox® could be rapidly performed. Unfortunately, it was not possible to show the benefits of modified Hayman suture technique with 2-Monodiox® because various factors about performing UCSs were not investigated (e.g., time required to perform UCSs, the amount of bleeding after UCSs, surgeons’ opinion) and neither compared these results with other sutures and needles; thus, this was the limitation of our study. Instead, we considered that our video file could show the simplicity of 2-Monodiox® (Additional file 1). Our literature review showed that only three dedicated needles were used for UCSs: No. 1 poliglecaprone 25 suture (W3709; Ethicon Endo-Surgery, Inc.), which was developed to perform the B-Lynch technique [16, 17], and a customized sizable curved needle, as reported by Yano et al., which was developed to perform the MY suture technique.^18^ W3709 was recommended by B-Lynch et al. as the most suitable material for the B-Lynch suture. However, our literature review revealed that about 80% of the included studies used Polyglactin 910 and Catgut sutures (data not shown), and W3709 was used in three reports (including our study). Zhang Z et al. considered that this is because W3709 was not available in most hospitals [29].

Our study has two strong biases. Firstly, it is the selection bias of our study. Our study focused on the efficacy of different needles and sutures for UCSs to the uterine body due to atonic bleeding; therefore, most of the high risk and severe PPH patients such as placenta previa and placenta previa complicated with placenta accreta cases, have not been included. These selection biases resulted in low incidence of severe PPH. Secondly, it is the lack of multivariate analysis to analyze variable factors whether these factors affect uterine preservation rate (B-lynch, Hayman, needle, parity, the number of previous cesarean delivery, etc.). Also, small sample sizes were included in this study that did not allow us to perform a multivariate analysis, which is the strong bias of this study.

The strength of this study was that it is the first study comparing needles and sutures for UCSs and the first report of UCSs using a dedicated blunt straight needle. The limitation to this study is the lack of data regarding the benefits of a dedicated blunt straight needle and suture for UCSs, and it was not possible to investigate the differences in the efficacy of each needle and suture to assess the uterine preservation rate among the different UCS types.

## Conclusion

The present retrospective study focused on sutures and needles used for UCSs. Three types of sutures were compared and our data showed that No. 0 sutures should not be used for B-Lynch suture. Our dedicated blunt straight needle with No. 2 polydioxanone sutures (2-Monodiox®) could perform modified Hayman suture with similar uterine preservation rates when performed with B-Lynch technique with No. 1 sutures. Our future studies are expected to show the efficacy and simplicity of 2-Monodiox®.

## Abbreviations

PPH: Postpartum hemorrhage
UCS: Uterine compression suture
